# Phenome-Wide Association Study to Explore Relationships between Immune System Related Genetic Loci and Complex Traits and Diseases

**DOI:** 10.1371/journal.pone.0160573

**Published:** 2016-08-10

**Authors:** Anurag Verma, Anna O. Basile, Yuki Bradford, Helena Kuivaniemi, Gerard Tromp, David Carey, Glenn S. Gerhard, James E. Crowe, Marylyn D. Ritchie, Sarah A. Pendergrass

**Affiliations:** 1 Biomedical and Translational Informatics Program, Geisinger Health System, Danville, Pennsylvania, United States of America; 2 Center for Systems Genomics, Department of Biochemistry and Molecular Biology, The Huck Institutes of the Life Sciences, The Pennsylvania State University, University Park, Pennsylvania, United States of America; 3 The Sigfried and Janet Weis Center for Research, Geisinger Health System, Danville, Pennsylvania, United States of America; 4 Division of Molecular Biology and Human Genetics, Department of Biomedical Sciences, Faculty of Medicine and Health Sciences, Stellenbosch University, Tygerberg, South Africa; 5 Department of Medical Genetics and Molecular Biochemistry, Temple University School of Medicine, Philadelphia, Pennsylvania, United States of America; 6 The Vanderbilt Vaccine Center, Vanderbilt University, Nashville Tennessee, United States of America; Kunming Institute of Zoology, Chinese Academy of Sciences, CHINA

## Abstract

We performed a Phenome-Wide Association Study (PheWAS) to identify interrelationships between the immune system genetic architecture and a wide array of phenotypes from two de-identified electronic health record (EHR) biorepositories. We selected variants within genes encoding critical factors in the immune system and variants with known associations with autoimmunity. To define case/control status for EHR diagnoses, we used International Classification of Diseases, Ninth Revision (ICD-9) diagnosis codes from 3,024 Geisinger Clinic MyCode^®^ subjects (470 diagnoses) and 2,899 Vanderbilt University Medical Center BioVU biorepository subjects (380 diagnoses). A pooled-analysis was also carried out for the replicating results of the two data sets. We identified new associations with potential biological relevance including SNPs in tumor necrosis factor (*TNF*) and ankyrin-related genes associated with acute and chronic sinusitis and acute respiratory tract infection. The two most significant associations identified were for the *C6orf10* SNP rs6910071 and “rheumatoid arthritis” (ICD-9 code category 714) (p_*METAL*_ = 2.58 x 10^−9^) and the *ATN1* SNP rs2239167 and “diabetes mellitus, type 2” (ICD-9 code category 250) (p_*METAL*_ = 6.39 x 10^−9^). This study highlights the utility of using PheWAS in conjunction with EHRs to discover new genotypic-phenotypic associations for immune-system related genetic loci.

## Introduction

Autoimmune diseases affect about 5% of the population and can lead to chronic inflammation targeting specific tissues [1]. The most common autoimmune diseases, such as rheumatoid arthritis (RA), multiple sclerosis, and type 1 diabetes mellitus (T1DM), have overlapping clinical, epidemiological and therapeutic features, but their genetic underpinnings and pathogenesis are still not fully understood [2]. Genome Wide Association Studies (GWAS) have discovered over 200 genetic loci associated with autoimmune diseases [2], elucidating biological pathways and potential drug targets for autoimmune disorders [3]. Comparison of results across GWAS shows a series of single nucleotide polymorphisms (SNPs) associated with multiple autoimmune diseases, suggesting the existence of variance in immune traits and pleiotropy [3]. For example, multiple genetic variants that reside within the region encompassing the human leukocyte antigen (HLA) system have been associated with several autoimmune diseases [4]. Although GWAS have identified multiple autoimmune disease susceptibility loci, the biological relationship between genetic variation within these loci and disease status has not been well characterized.

While genetic variation in immune function and inflammation contributes to susceptibility to autoimmune conditions, this variation may also impact a variety of other diseases and diagnoses. The immune system serves as a major defense network in fighting disease and infection. Genetic variation in immune function has been found to contribute to disease susceptibility in multiple classes of disorders [3]. For example, monocyte-specific expression quantitative trait loci (eQTLs) have been identified for genetic variants associated with neurodegenerative disorders such as Parkinson’s and Alzheimer’s diseases [5]. As a manifestation of immune function, inflammation also plays an important role in conditions beyond contagious or autoimmune diseases. For instance, inflammation has been implicated in multiple disorders including vascular diseases such as atherosclerosis [6] and congestive heart failure [7], neuropsychiatric diseases like autism [8], as well as metabolic traits and disorders such as obesity [9] and type 2 diabetes (T2DM) [10].

To examine potential associations across many phenotypes, Phenome-wide association studies (PheWAS) have been developed as a complementary approach to GWAS, using all available phenotypic information and genetic variation in order to estimate the association between genotype and phenotype [11]. PheWAS are dependent on comprehensive phenotypic information on large numbers of individuals; PheWAS to date have used electronic health record (EHR) International Classification of Diseases (ICD-9) billing codes to define case-control statuses for multiple diagnoses [12], data from epidemiological studies with hundreds to thousands of phenotypic measurements [13][11], as well as clinical trials data [14]. The PheWAS framework of evaluating the association between a wide array of phenotypes and markers permits the study of pleiotropy, compared to the GWAS framework of investigating association between a single trait and genetic markers, except when comparing results from multiple separate GWAS [15]. In this PheWAS, we used variants in immune-related genes which provided an opportunity to explore the association between immune system SNPs and phenotypes beyond specific autoimmune and immune system traits, such as diagnoses that may have an immune system involvement but are not specifically classified as an autoimmune/immune system trait.

The goal of this study was to identify associations between selected SNPs with known or possible associations with autoimmune disease and the immune system and a variety of diagnoses, evaluating and contrasting results across two separate EHR systems. We performed our PheWAS analysis using SNPs within genes encoding critical factors for the immune system and SNPs with known associations with autoimmunity, including a series of SNPs also found on ImmunoChip, an array designed by investigators of 11 autoimmune and inflammatory diseases [16,17]. To explore associations between these SNPs and diagnoses, we used ICD-9 diagnosis codes to define case/control status from two sites within the Electronic Medical Record and Genomics (eMERGE) Network: Geisinger MyCode^®^ and Vanderbilt BioVU. Highly significant results were investigated within the individual datasets, and replication of associations was also sought across the two different bio-repositories. The results of this study also demonstrate cross-phenotype associations that may be due to pleiotropy and identified complex networks that exist between immune related genetic variants and many different diagnoses.

## Methods

### Data Sets

We used de-identified EHR biorepository data linked to genotypic data and ICD-9 diagnosis code data from two sites in the eMERGE Network: Geisinger Health System’s MyCode^®^ and Vanderbilt University Medical Center’s BioVU [18]. The MyCode dataset had a total of 3,024 individuals and the BioVU dataset had 2,899 individuals available for the study with *both* phenotypic and genotypic data (Table 1). Because a majority of subjects in MyCode^®^ were of European ancestry (EA), we selected only EA subjects to seek replication with the BioVU data [19].

**Table 1:** Summary of Data Sets Used for the Study.

EHR Site	Total Sample Size	% Male	Median Age (in decade)	Case Size Range	Genotyping Platform	Number of SNPs Pre-imputation	Number of SNPs Post-imputation	Number of SNPs after filtering	Number of Diagnosis Codes
Geisinger MyCode^®^	3024	53.0	40	Min = 11; Max = 1898; Median = 32	Illumina Human OmniExpress	729,078	38,054,243	95,448	477
Vanderbilt BioVU	2899	45.4	60	Min = 11; Max = 1056; Median = 31	Illumina 660	558,980	38,041,351	87,690	380

**Fig 1:**
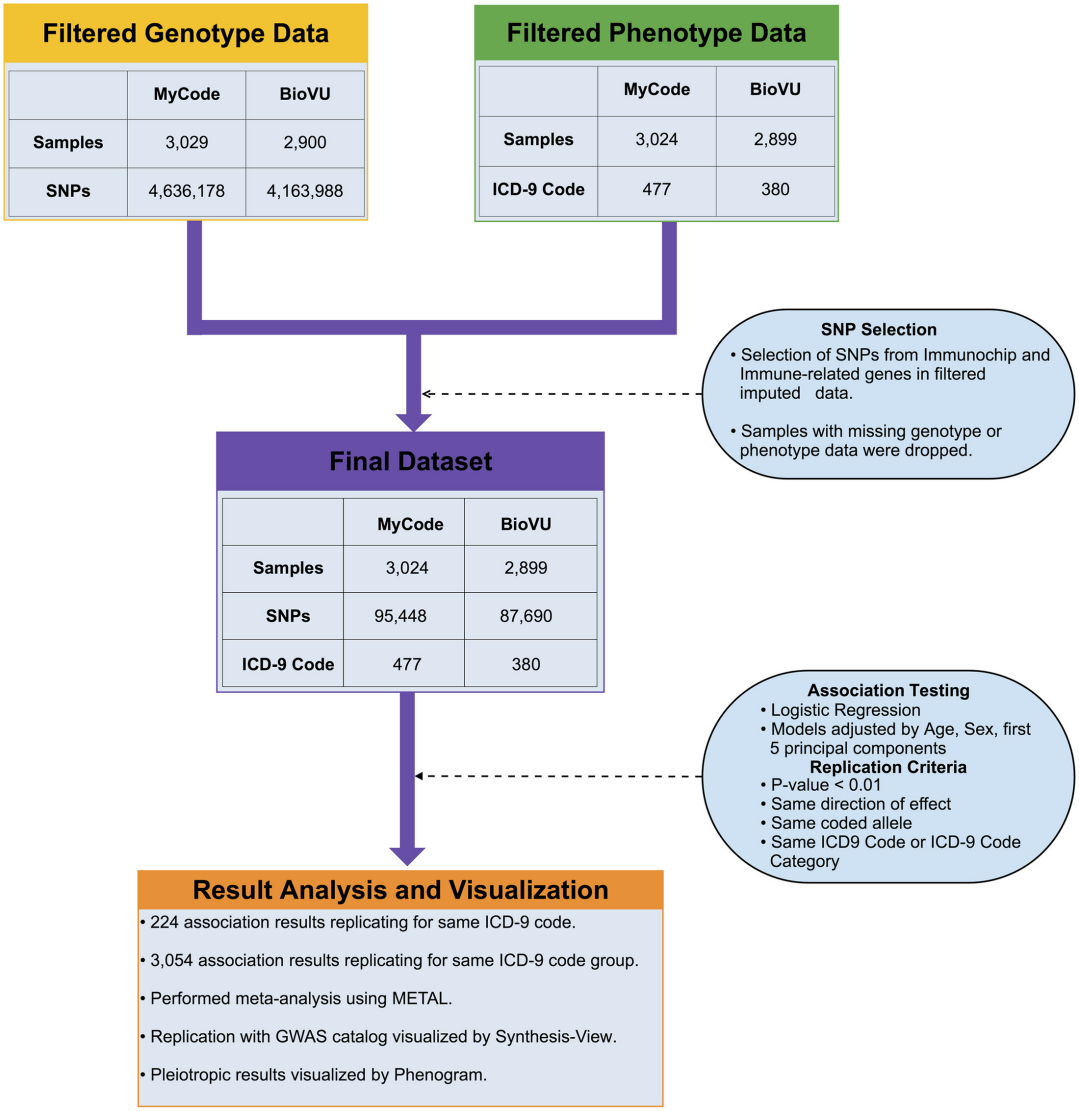
Overview of PheWAS with Immune Variants. This flow chart provides an overview of the steps taken to perform PheWAS between immune variants and ICD-9 diagnosis codes. The final testing dataset (purple) was formed by selecting SNPs from our array data that also exist on Immunochip and/or are within immune-related genes (yellow) and removing samples with missing genotypic or phenotypic data (green). Comprehensive associations were calculated between all final dataset SNPs and ICD-9 code based case/control status using logistic regression, with all models adjusted for age, sex and first five principal components. Replication was sought following both an exact ICD-9 code and a category ICD-9 code approach following the specified criteria. Pooled analysis was performed for both approaches using METAL. See S1 Fig for the full workflow from imputation through quality control, association testing, and replication for this study.

For additional information on the study design, see Figs 1 and S1.

The Geisinger biorepository has had both general and targeted recruitment for specific diseases, such as obesity and abdominal aortic aneurysms (AAA). BioVU has consented using an opt-out approach, where individuals with discarded blood may or may not be added to the biorepository unless they indicate they would like to opt-out of BioVU [20]. Thus BioVU has no pre-selection for individuals with a specific disease phenotype.

### Genotyping, Imputation & Quality Control

We summarize the genotyping, imputation, and quality control procedures in S1 Fig. Geisinger MyCode^®^ subjects were genotyped using the Illumina HumanOmniExpress-12 v1.0 array, a total of 729,078 SNPs. Genotyping of BioVU subjects was performed using the Illumina 660 Quad array, a total of 558,590 SNPs. We used imputation for improved genomic coverage and overlap between datasets of immune related variants. We performed imputation using the IMPUTE2 algorithm [21] after phasing with SHAPEIT2 [22] using the 1,000 Genomes cosmopolitan reference panel, resulting in a total of 38,054,243 SNPs in 3,111 samples for MyCode^®^ and 38,041,351 SNPs in 3,375 samples for BioVU [23].

Genotype Quality Control (QC) procedures were performed prior to association testing using the R programming statistical package [24] and PLINK software [25]. QC was performed on each dataset separately. The first step was to filter out the SNPs with poor imputation quality; SNPs with imputation quality scores > 0.9 were used for further analyses. Data were filtered further for 99% genotype and sample call rates and minor allele frequency (MAF) threshold of 1%. Also, related samples were removed using Identity by Descent (IBD) kinship coefficient estimates. We also performed principal component analysis (PCA), determining principle components to use to correct for population differences within the EA of these datasets, as association results for immune system genes in particular can be particularly affected by population substructure. After QC, the genotypic data consisted of 4,636,178 SNPs and 3,029 samples from MyCode^®^ and 4,163,988 SNPs and 2,900 samples from BioVU.

### Phenotype Data

To define case-control status for each ICD-9 code, a MySQL database was used to assemble the phenotypic data, consisting of 6,525 ICD-9 codes from the MyCode^®^ dataset and 1,206 ICD-9 codes from BioVU. A case was defined as an individual with more than three instances of a specific ICD-9 code. Controls were defined as individuals not meeting the case criteria. More than ten case subjects were required for inclusion of a diagnosis in our study. Using these criteria there, were 3,024 samples and 477 ICD-9 codes in MyCode^®^, and 2,899 samples and 380 ICD-9 codes in BioVU. For replication of results across the two studies, there were a total of 50 exact ICD-9 code matches (i.e. 3, 4 and 5 digit ICD-9 code) across both datasets, and a total of 186 ICD-9 category (i.e. three digit ICD-9 code) matches across both datasets.

### Selection of SNPs for the Study

While we used genetic data from Illumina arrays, we focused on SNPs related to the immune system, and chose from our array data SNPs present on ImmunoChip (Illumina) or known to be involved in the immune system within a specific set of genes (see S1 Table for a list of these 34 genes). The ImmunoChip is a custom genotyping array designed by Illumina with 195,806 SNPs for performing deep replication of associations with major autoimmune and inflammatory diseases including fine mapping of GWAS loci covering 11 major autoimmune diseases (*e*.*g*., T1DM, autoimmune thyroid disease, celiac disease and multiple sclerosis), seronegative diseases (*e*.*g*., ulcerative colitis, Crohn's disease, and psoriasis), and rheumatic diseases (*e*.*g*., RA, ankylosing spondylitis and systemic lupus erythematosus) [17]. Also included on ImmunoChip are all the previously confirmed GWAS SNPs for which probes could be designed using data from the 1,000 Genomes Project [16]. From this study, we selected only SNPs from our genome-wide array data that were on the ImmunoChip array or within the genes we identified for involvement in the immune system. The SNP filtering process is shown in Fig 1.

Biofilter was the tool used to generate the list of SNPs for association testing. Biofilter is a software tool with an extensive database containing biological knowledge from publicly available repositories of biological data that can be used to annotate genomic information, as well as filter genomic information based on specific criteria [26,27]. First Biofilter 2.1 was used to annotate the post-QC SNPs of this study with gene information for any SNPs within Entrez-defined gene boundaries. Then Biofilter was used with the genotypic data from each EHR site to filter SNPs, maintaining only those SNPs matching within the 34 genes selected for their known involvement in the immune system (S1 Table). The SNP filtering step resulted in 95,448 SNPs from MyCode^®^ and 87,690 SNPs from BioVU, with a total of 76,861 SNPs overlapping across the two datasets available for association testing (Table 1).

### Association Testing and Identifying Replication

In both datasets, separately, associations were calculated using logistic regression with models adjusted for sex, age, and the first five principal components. In the MyCode^®^ dataset, using logistic regression, the strength and significance of associations were evaluated between 95,448 SNPs and 477 clinical diagnoses. There were a total of 366,468 associations with p < 0.01. In the BioVU dataset, association testing was performed on 87,690 SNPs and 380 phenotypes. There were a total of 261,346 associations with p<0.01. We also compared our results to a Bonferroni corrected p-value threshold. A LD pruning approach was used to account for correlation between the SNPs and identified independent SNPs at r^2^ = 0.3 [28]. For MyCode and BioVU association testing, the Bonferroni threshold was 4.73 x 10^−9^ [0.05/(22,138 x 477)] and 6.07 x 10^−9^ [0.05/(21,673 x 380)], respectively. There was only one result that passed the conservative Bonferroni threshold in MyCode and none of the results passed threshold in BioVU.

A MySQL database was used to organize all association results. This included inspection of results in single datasets where the ICD-9 code existed only in one or the other dataset. S2 Table shows the results of MyCode^®^ and BioVU with p<1x10^-4^, where we could not seek replication across both datasets, as the ICD-9 codes were only specific to each study. For replication we used two approaches. In the first approach, association results replicating across both datasets for the same SNP and *exact ICD-9 diagnosis code*, with same direction of effect of the association were used. In the second approach, we also used the database to seek replication across both datasets, for the same SNP and *ICD-9 code category*, with the same direction of effect of the association. ICD-9 codes classify diagnoses; there are three digit ICD-9 codes that specify disease categories (e.g. code 405 for “secondary hypertension”) that can be further subdivided using multiple four or five digit sub ICD-9 codes (e.g. 405.1 for “benign secondary hypertension”, 405.11 “benign renovascular hypertension”). Wide variation exists across institutions in the way specific ICD-9 codes are applied, although three digit ICD-9 categories are used more consistently for diagnoses from institution to institution. We therefore analyzed results based on replication requiring the *exact* ICD-9 code used in the association (*i*.*e*. three, four, or five digit sub ICD-9 codes), as well as evaluating results based on replication requiring only the same three digits of the ICD-9 code category. S3 and S4 Tables show all of the results where the criteria for replication were met for the same ICD-9 code, and for the same ICD-9 code category, respectively.

We also annotated SNPs in replicating associations between BioVU and MyCode^®^ with information from the NHGRI-EBI GWAS catalog [29,30] and GRASP [31,32], thus identifying any previously reported associations for these SNPs. We used a p-value threshold of 1x10^-5^ (default for NHGRI-EBI GWAS catalog) on the associations reported in both the sources.

### Focusing on Immune System and Autoimmune Traits

We further explored results for associations with phenotypes/diagnoses more closely linked to the immune system or autoimmune disease. Thus, we filtered the results presented in S4 Table (all results where the criteria for replication were met for the same ICD-9 code category) for only ICD-9 categories related to the immune system or autoimmune disease by having three individuals identify any ICD-9 categories for removal that were too broad (such as “general symptoms, not otherwise specified”, etc.), cancer related diagnoses, and diagnoses clearly related to accident or surgery (such as “hypothyroidism due to ablation”). In this way, we retained ICD-9 codes describing autoimmune reactions or clearly influenced by immune system variation. Table 2 lists the ICD-9 categories and descriptions selected through this process, as well as the genes in which the variants are located. This process resulted in 409 associations (S5 Table).

**Table 2:** Immune-Related ICD-9 categories selected for further analysis.

ICD-9 General Classification	ICD-9 Code Category (Code: Category Description)	Nearest Genes
Endocrine, nutritional and metabolic diseases, and immunity disorders	250: Diabetes mellitus, Type 1	*LOC645266*, *MTCO3P1*, *HLA-DRA*, *HLA-DQB2*, *HLA-DOB*, *DDC*, *DDC*, *LOC100129427*, *HLA-DMA*, *HCG23*, *C6orf10*, *MIR588*, *CAST*, *EPHA5*, *LOC645321*, *SERPINB11*, *KC6*, *FLJ30679*, *PRELID1P1*, *RPS4XP9*, *THEMIS*, *HIST1H1T*
	273: Disorders of plasma protein metabolism	*LOC100996339*, *ESRRG*
Diseases of the nervous system	331: Other cerebral degenerations	*QRSL1*
	340: Multiple sclerosis	*MYT1L*, *IRF4*
	357: Inflammatory and toxic neuropathy	*GTDC2*, *RAB38*, *LOC100129160*
Diseases of the sense organs	373: Inflammation of eyelids	*USH2A*, *MAP4K4*
Diseases of the respiratory system	461: Acute sinusitis	*CNTNAP2*, *ANKS1A*
	465: Acute upper respiratory infections of multiple or unspecified sites	*RPL23AP54*, *ZZEF1*, *ANK3*
	466: Acute bronchitis and bronchiolitis	*CLSTN2*, *LOC100129949*
	472: Chronic pharyngitis and nasopharyngitis	*DAP3P2*, *DCAF17*, *LOC100287243*, *METTL8*, *TAF3*, *KIAA1217*
	473: Chronic sinusitis	*ANK3*, *TRPS1*, *KSR1*, *RBM17*
	477: Allergic rhinitis	*KCNK3*,*VAV3-AS1*,*PADI4*,*MED13*,*KIRREL3*,*TOX*,*FTLP7*
	482: Other bacterial pneumonia	*TLR6*, *LOC645481*
	491: Chronic bronchitis	*SDC4*,*RPS2P9*, *SYS1-DBNDD2*,*PITX2*,*SYS1*,*SYS1-DBNDD2*,*XCR1*
	492: Emphysema	*GABRA4*
	493: Asthma	*TPD52L1*, *CAST*, *WDR11*
	515: Postinflammatory pulmonary fibrosis	*SLC16A10*,*FRMD6-AS2*,*LOC100506923*,*ADIPOR1*
Diseases of the digestive system	556: Ulcerative colitis	*BTF3L4P3*
	571: Chronic liver disease and cirrhosis	*RSBN1*, *RFX3*, *CTIF*, *PHTF1*
	577: Diseases of pancreas	*LCE1B*, *NTRK3-AS1*
Diseases of the genitourinary system	584: Acute renal failure	*FAM205B*, *LACC1*, *DGKZP1*, *E2F3*, *FAM205A*
	585: Chronic kidney disease	*STXBP4*, *LOC100996324*, *CCDC148*, *RPL13AP25*, *BMP2*, *RRP15*
	586: Renal failure	*PLK2*, *LOC100507162*
	595: Cystitis	*SDK1*, *DNTTIP2*
Diseases of the skin and subcutaneous tissue	692: Contact dermatitis and other eczema	*MB21D2*
	695: Erythematous conditions	*SRRM4*, *PRDM15*, *VDR*, *INTS6*, *FGFR3P3*
Diseases of the musculoskeletal system and connective tissue	714: Rheumatoid arthritis and other inflammatory polyarthropathies	*MTCO3P1*, *HLA-DRA*, *NOTCH4*, *TNIP1*, *IL6*, *HLA-DRB1*, *C6orf10*, *HLA-DRB9*
	715: Osteoarthrosis and allied disorders	*BRD2*, *SEMA6A*, *FLJ42102*, *RNY4P22*, *TNFSF8*, *KIAA1919*, *BACH2*, *AKT3*, *SDHAP3*, *ZBTB38*, *CAMK1G*, *PRDM1*, *U2SURP*, *RETNLB*, *ANXA6*
	716: Other and unspecified arthropathies	*IL23R*, *GPX3*, *TRNAS13*, *ZNF192P2*, *AGPAT4*, *QKI*, *ENTPD7*, *GJD4*
	719: Other and unspecified disorders of joint	*TCF4*, *LOC100288337*, *TBX3*

### Visualization Tools

We used Synthesis-View [33], PhenoGram [34], and Cytoscape [35], to visualize the results. Synthesis-View was used to visualize the SNP-phenotype associations and to plot associations matching previously reported associations in the NHGRI-EBI GWAS catalog and GRASP. PhenoGram was used to visualize potentially pleiotropic SNPs by creating a chromosomal ideogram with lines denoting SNP locations and colored circles depicting phenotypes associated with those SNPs.

We also used Cytoscape 3.0 to visualize network diagrams from the significant PheWAS results. To produce the Cytoscape plots, we first used Biofilter to annotate the PheWAS result SNPs with the gene, or the closest gene to the SNP. We then used Biofilter to obtain the gene annotations from Kyoto Encyclopedia of Genes and Genomes (KEGG) pathways [36].

### Functional Annotation

To obtain functional information, we annotated the SNPs used in this study with HaploReg V2 [37] and SNP and CNV Annotation Database (SCAN) [38]. HaploReg provides functional annotation of SNPs within LD blocks, and includes information on chromatin state in multiple cell types, regulatory motif alterations and sequence conservation. SCAN provides information from eQTL experiments with a list of genes whose expression is affected by the given SNP in Caucasian (CEU) and Yoruba (YRI) populations. We used expression data specific to the CEU population from SCAN.

## Results

### PheWAS in Two EHR Datasets Using ICD-9 Codes

Fig 1 provides an overview of our study to identify comprehensive associations between immune system related variants and ICD-9 based case/control diagnoses within MyCode^®^ and BioVU data (further details available in Methods). S1 Fig shows the full workflow, from imputation through quality control, association testing and replication for this study. Table 1 provides a summary of the datasets used. Only subjects of EA with both phenotypic and genotypic data within these sites were selected for discovery and replication analyses.

Evaluating the two EHR datasets separately yielded a total of 366,468 associations (p < 0.01) between SNPs and ICD-9 codes in the MyCode^®^ dataset and 261,346 associations (p < 0.01) in the BioVU dataset (see details on datasets in Methods). The most significant association in MyCode^®^ passing our Bonferroni threshold of 4.73 x 10^−9^ was between rs41272317 in the *ACAD11* gene on chromosome 3 and “symptoms concerning nutrition metabolism and development” (ICD-9 code 783.21), with p = 8.15 x 10^−12^ (β = 2.68, cases = 45, controls = 2,979). In the BioVU PheWAS results, the most significant association result was between SNP rs870769 on chromosome 8 and “transient cerebral ischemia” (ICD-9 code 435), with p = 7.41 x 10^−8^ (β = 2.2, cases = 32, controls = 2,861), which did not pass our Bonferroni threshold. While these are most significant associations within each dataset independently, they did not replicate across both MyCode and BioVU.

Geisinger’s genotyped cohort from MyCode^®^ had a large number of cases with AAA or obesity, warranting further exploration for immune variation related to AAA and obesity. Inflammatory processes have been implicated in AAA as well as obesity [39]. We found an association within MyCode between “abdominal aortic aneurysm without mention of rupture” (ICD-9 code 441.4) and rs11084402 on chromosome 19 (p = 2.22 x 10^−5^, β = 0.36, cases = 778, controls = 2,246). There were no associations for AAA in the BioVU dataset.

We further explored results with diagnosis codes meeting the criteria for inclusion, but for associations where we could not seek replication because that ICD-9 code was not present in the other dataset. There were a total of 50 exact ICD-9 code matches across both datasets, and a total of 186 exact ICD-9 category matches across both datasets, which placed limitations on seeking replication across the two sets. For the results where we could not seek replication due to the ICD-9 codes not being present in the other dataset, we focused our attention on association results with the highest case numbers (hundreds to thousands of cases) with p < 1x10^-4^. While this p-value cutoff is less stringent than our Bonferroni cutoff, we chose a more exploratory p-value cutoff focused on the most highly suggestive and powered associations from the PheWAS. S2 Table lists these results for MyCode^®^ and BioVU with p<1x10^-4^. In MyCode^®^, we found associations between SNPs and metabolic disorder traits, including with “essential primary hypertension” (ICD-9 code 401.9), “other and unspecified hyperlipidemia” (ICD9-code 272.4), and “morbid obesity” (ICD-9 code 278.01). Interestingly, there were related metabolic disorder traits in BioVU, but these did not replicate the MyCode^®^ results. For example, among the highest case numbers in BioVU for p<1x10^-4^ there were associations between SNPs and diagnoses including “essential hypertension” (ICD9-code 401), and “disorder of lipoid metabolism” (ICD-9 code 272).

### PheWAS Results with Replication

To identify further robust associations, we also sought replication of results across the two datasets using two approaches: seeking results for the same SNP and *same ICD-9 code category (i*.*e*. truncating the ICD-9 code for each case/control status to the three digit ICD-9 code) with p<0.01 and the same direction of association, and also seeking results for the same SNP and *exact ICD-9 code* (*i*.*e*. the exact ICD-9 case/control status for each association, varying from three to five digits) with p<0.01 and the same direction of association (Fig 1). There were a total of 224 associations with exact ICD-9 code replication across the two datasets with p<0.01, for the same SNP, coded allele, and direction of effect (S3 Table). Of the 224 replicated associations with the exact same ICD-9 code, the most significant association in BioVU that replicated in MyCode^®^ was between “soft tissue disorders” (ICD-9 code 729.1) and the *PLA2G2E* SNP rs1108975 with p_BioVU_ = 3.29 x 10^−6^ (Case-Control = 43/2,853), replicating in the MyCode^®^ data with p_MyCode_ = 3.29 x10^-3^ (Case/Control = 136/2,888). The most significant association in the MyCode^®^ data, replicating in the BioVU dataset was between SNP rs11869607 and the diagnosis “deficiency anemias” (ICD-9 281.1) with p_MyCode_ = 5.98 x 10^−5^ (Case/Control = 21/3003) and in BioVU p_BioVU_ = 3.07 x 10^−3^ (Case/Control = 16/2,882).

We had a total of 3,054 results for the same SNP, coded allele, and direction of effect, when the replication criterion was based on requiring the same ICD-9 code category, (S4 Table) and association between *F5* SNP rs6427196 and “pulmonary embolus” (ICD-9 code category 453) was most significant with p_MyCode_ = 1.3 x 10^−7^ (Case/Control_*MyCode*_ = 53/2,970) and p_BioVU_ = 5.72 x 10^−3^ (Case/Control_*BioVU*_ = 63/2,834).

We used METAL [40] to perform a pooled-analysis for both sets of results meeting our criteria for replication across BioVU and MyCode^®^ (S3 and S4 Tables). The most significant of these associations was between the diagnosis “myalgia and myostosis” (ICD-9 code 729.1) and the *PLA2G2E* SNP rs1108975 with p_*METAL*_ = 8.99 x 10^−8^ (Case/Control_*MyCode*_ = 136/2,888, Case/Control_*BioVU*_ = 43/2,853). Of the 3,054 results meeting our PheWAS replication criteria for the same ICD-9 code category, the most significant association was between the diagnosis “rheumatoid arthritis and other inflammatory polyarthropathies” (ICD-9 code category 714) and *C6orf10* SNP rs6910071 with a meta-analysis p_*METAL*_ = 2.58 x 10^−9^ (Case-Control_*MyCode*_ = 60/2,964, Case-Control_*BioVU*_ = 81/2,818). Another top association signal was between the *ATN1* SNP rs2239167 and “diabetes mellitus, type 2” (ICD-9 category code 250) with a p_*METAL*_ = 6.39 x 10^−9^ (Case-Control_*MyCode*_ = 23/3,001, Case-Control_*BioVU*_ = 41/2,858).

### Matching Previously Reported GWAS Results

Results replicating in both biorepositories using the exact ICD-9 code and ICD-9 category-based PheWAS were evaluated for any matches to SNPs with previously reported associations for the same phenotypes with significance of p-value<1x10^-5^ in the NHGRI-EBI GWAS catalog and GRASP. We found that GRASP included GWAS results from many more studies than the NHGRI-EBI catalog and most association in NHGRI-EBI were also reported in GRASP. However, we report SNP associations from previous GWAS if it is reported in either NHGRI-EBI catalog or GRASP.

Of the SNPs in the 224 exact ICD-9 code replicating associations, we found a total of 10 SNPs with phenotypic associations also previously reported in existing GWAS. However, none of these SNPs were associated with the same phenotypes in our study compared to existing GWAS. For example, the *PARD3B* SNP rs1207421 is reported in the GWAS catalog to be associated with “knee osteoarthritis” (reported GWAS p = 6 x 10^−6^) [41]. In our study this SNP had a novel association with “scar conditions and fibrosis of skin” (ICD-9 code; 709.2), and was not associated with osteoarthritis.

**Fig 2:**
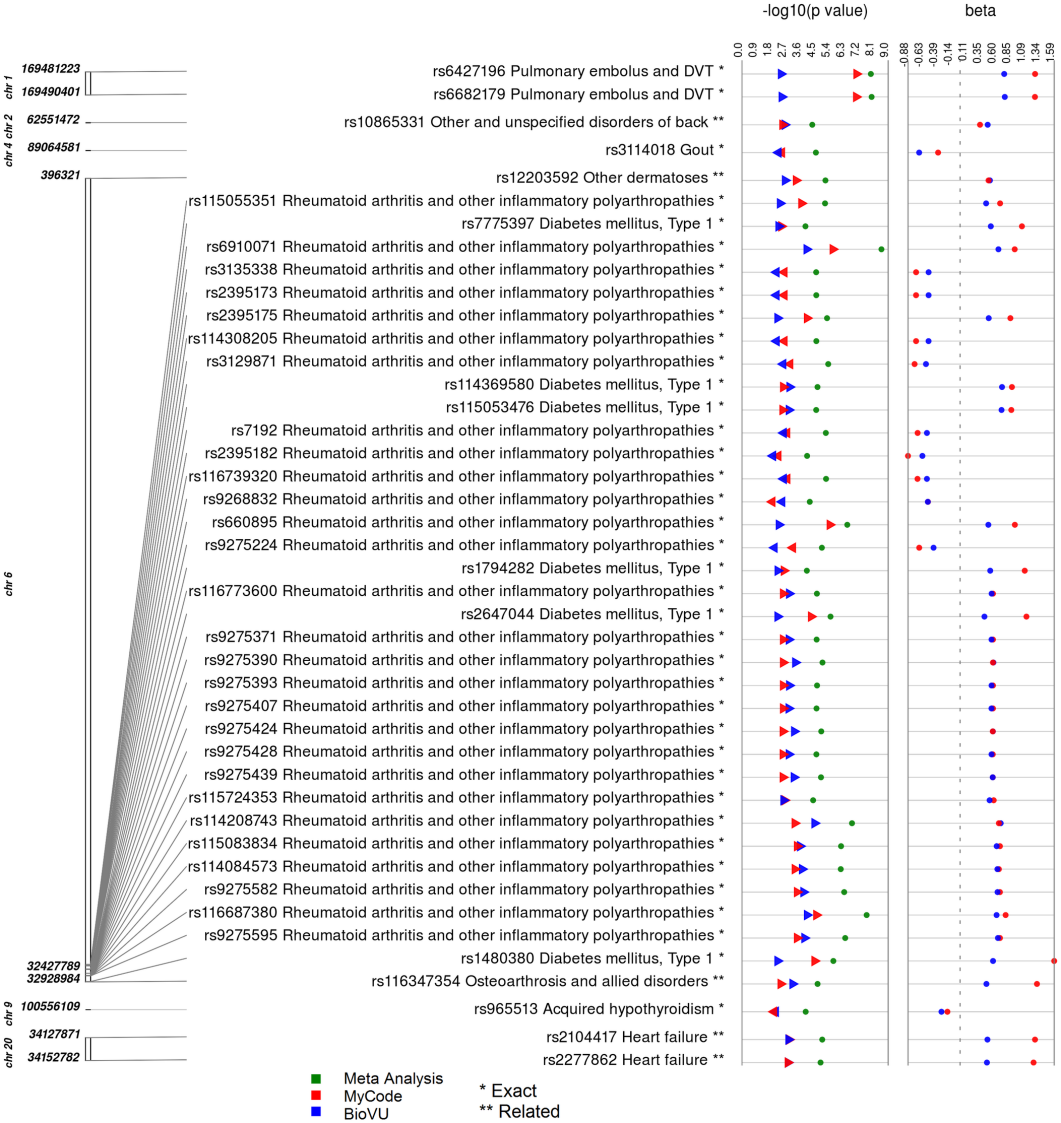
Synthesis view plot showing PheWAS results replicating across MyCode^®^ and BioVU that have previously reported associations. The first track is the chromosomal location for each SNP. The next column lists the SNP identifier, the phenotype associated in our study, and the reported GWAS trait (p<10^−5^). Results representing exact matches with the NHGRI-EBI GWAS catalog and GRASP are annotated with a single asterisk and the closely related traits are represented with a double asterisk. Blue symbols represent results from MyCode^®^, red symbols represent results from BioVU and green symbols are the pooled analysis results obtained using the program METAL.

In our results meeting the PheWAS criteria for replication in both datasets for the same ICD-9 code category, a total of 284 SNPs were also represented in either NHGRI-EBI GWAS catalog or the GRASP database. A total of 42 SNP-phenotype pairs matched identical associations reported in the GWAS catalogs, and five results had a diagnosis closely related to the phenotype reported in the GWAS catalogs. A few top SNP-phenotype pairs matching previously reported associations include the *C6orf10* SNP rs6910071 associated in our study with “rheumatoid arthritis and other inflammatory polyarthropathies” and reported previously to be associated with RA [42–44]. Also, an *F5* SNP rs6427196 and pulmonary embolus/DVT association (p_*METAL*_ = 1.16 x10^-8^, Case/Control_*MyCode*_ = 53/2,970, Case/Control_*BioVU*_ = 63/2,834) that has been previously reported with venous thromboembolism [45,46,46,47]. A SNP rs2647044 downstream of *MTC03P1* associated with “diabetes mellitus type 1” (p_*METAL*_ = 7.94 x10^-7^, Case/Control_*MyCode*_ = 22/3,002, Case/Control_*BioVU*_ = 98/2,801) in our study, was previously reported to be associated in GWAS with T1DM [48] and RA [42]. Finally, there was an association between rs660895 and “rheumatoid arthritis and other inflammatory polyarthropathies” (p_*METAL*_ = 3.28 x 10^−7^, Case/Control_*MyCode*_ = 85/2,939, Case/Control_*BioVU*_ = 138/2,761), and this SNP has shown previous association with RA [43]. Fig 2 shows a plot of the replicating associations found for our ICD-9 code category PheWAS for SNPs matching the exact or closely related ICD-9 code category description in previously reported studies.

### Associations with Immune and Autoimmune Related Diagnoses

**Fig 3:**
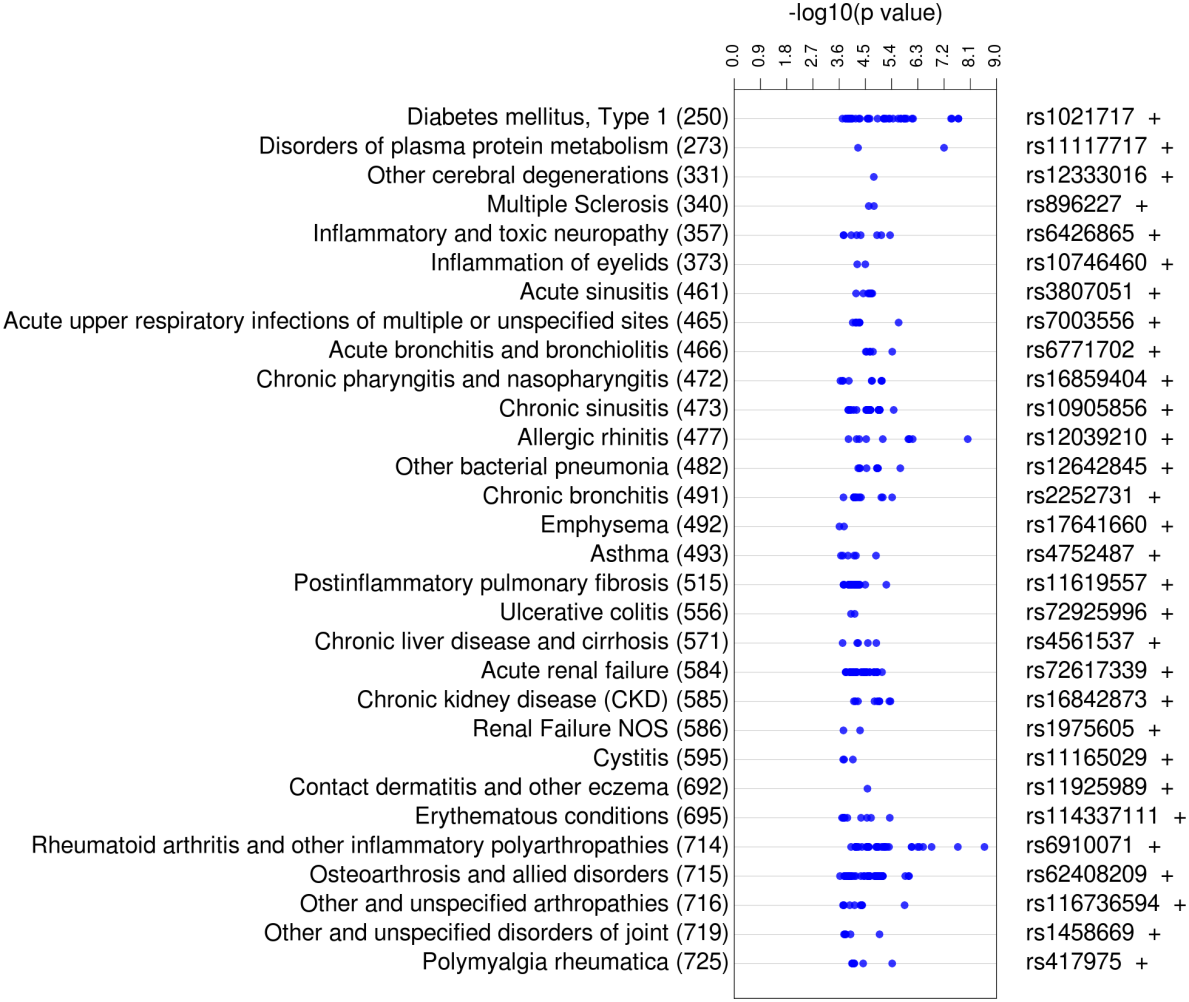
PheWAS View Plot of Meta-analysis Results with p<0.01 Replicating for the Same ICD-9 Category, Meeting Autoimmune and Immune-Related Diagnosis Criteria. The left track specifies the phenotype and ICD-9 Category code with which the SNP was associated. The next track indicates–log_10_(p-value) from the meta-analysis performed on all replicating SNPs with p<0.01. The last track indicates the SNP that had the most significant p-value, and the direction of effect of the association (+, positive; -, negative). The total number of associations between the SNPs and diagnoses was 409.

From the replicating category ICD-9 code results, we concentrated on the 441 SNP-ICD-9 code associations for immune- or autoimmune-related diagnoses, i.e. diagnoses more directly impacted by immune system variation. To assist developing a robust list of more specifically immune- or autoimmune-related ICD-9 codes, three separate researchers evaluated the selection of these ICD-9 code classes to reach consensus, resulting in 441 SNP-ICD-9 association results for further evaluation. In Table 2, we list the ICD-9 categories selected for this analysis as well as the genes with SNPs. S4 Table lists the association results meeting the criteria for significance and replication. Fig 3 presents the results for the 441 SNP-ICD-9 associations related to immune function.

Fig 3 shows the great diversity of the replicating results, ranging from autoimmune conditions, such as “rheumatoid arthritis” (RA) (ICD-9 code: 714), to contagious diseases, such as “bacterial pneumonia” (ICD-9 code: 482), to inflammatory diseases including “erythematous conditions” (ICD-9 code: 695). The diagnosis with the largest total number of replicating association results in Fig 3 was “osteoarthrosis” (ICD-9 code: 715) with 53 results in the MyCode^®^ and BioVU datasets. “Diabetes mellitus, type I” (TIDM) (ICD-9 code: 250) and “chronic sinusitis” (ICD-9 code: 473) each had 49 replicating results in both datasets.

Within this subset of results, the statistically most significant results include associations with RA, T1DM, and allergic rhinitis and osteoarthrosis. RA and T1DM are both common autoimmune diseases, and inflammation has been implicated in the pathogenesis of osteoarthrosis [49]. The most significant replicating association from the pooled analysis was between rs6910071 and the diagnosis RA with p_*METAL*_ = 2.58 x 10^−9^, as mentioned above this SNP has also been associated with RA in previous GWAS.

### Functional Annotation of Associated Variants within Genes

Of the 441 autoimmune and immune system related results, we next considered SNPs directly mapping to or within 50 kb of a gene to include promoter and regulatory regions, for potentially relevant genes. There were 233 associations of SNPs that mapped within genes. Herein, we again observed multiple variants mapped to genes with known relationships with associated phenotypes, particularly for RA and T1DM within the well-characterized HLA locus. This group included SNPs associated with T1DM within the *HLA-DMA*, and *HLA-DOB* genes, and SNPs associated with RA mapped to *HLA-DRB9* and *HLA-DRB1*. The SNP rs1480380 associated with osteoarthrosis and T1DM in our study is within 50 kb of *HLA-DMA*. Nine SNPs associated with T1DM in our study were in the *HLA-DRA* gene, and eight SNPs associated with RA also mapped to *HLA-DRA*. It is important to consider that the HLA region on chromosome 6 is highly polymorphic and there could be variability in HLA alleles due to population stratification. We only used EA individuals in this study, thus we expect less variation in the HLA region compared to a cohort across multiple ancestries. Further, we compared the MAFs of HLA region SNPs with 1000 Genomes EA population and the frequencies were very close.

### Functional Annotation of Associated Variants outside Protein-Coding Genes

Of the 441 replicating associations classified as autoimmune- or immune-related, there were 208 associations where SNPs mapped outside protein coding genes, with a total of 206 SNPs. We annotated the SNPs that did not map to genes with information on potential functionality using two public databases: HaploReg V2 and SCAN (S6 Table).

A total of 40 SNPs were associated with significantly altered gene expression in HapMap CEU lymphoblastoid cell lines in the SCAN database. The statistically most significant eQTL in SCAN for our 206 SNPs is rs2395182 and the expression of eight HLA locus genes as well as other genes including *RNASE2*, and *ZNF749*. In our study, this SNP was associated with “rheumatoid arthritis and other inflammatory polyarthropathies” (ICD-9 code: 714). This SNP is located 495 bp upstream of *HLA-DRA*. Another highly significant SCAN eQTL exists between the SNP rs1794282 and altered expression of *HLA-DQA1* and *HLA-DQA2*. In our study, SNP rs1794282 was associated with “type 1 diabetes” (ICD-9 code: 250).

We also used HaploReg to annotate the 206 SNPs that were outside gene boundaries, as well as any SNPs in LD (r^2^ > 0.8) with the original SNPs. Of these 206 SNPs, 134 had altered regulatory motifs in HaploReg and are likely to influence transcriptional regulation. Sixteen of the SNPs were reported to be strong enhancers in one cell type, and 47 SNPs are weak enhancers in one or more cell types, supporting the potentially functional role of these variants.

### Pleiotropy and Association Network

**Fig 4:**
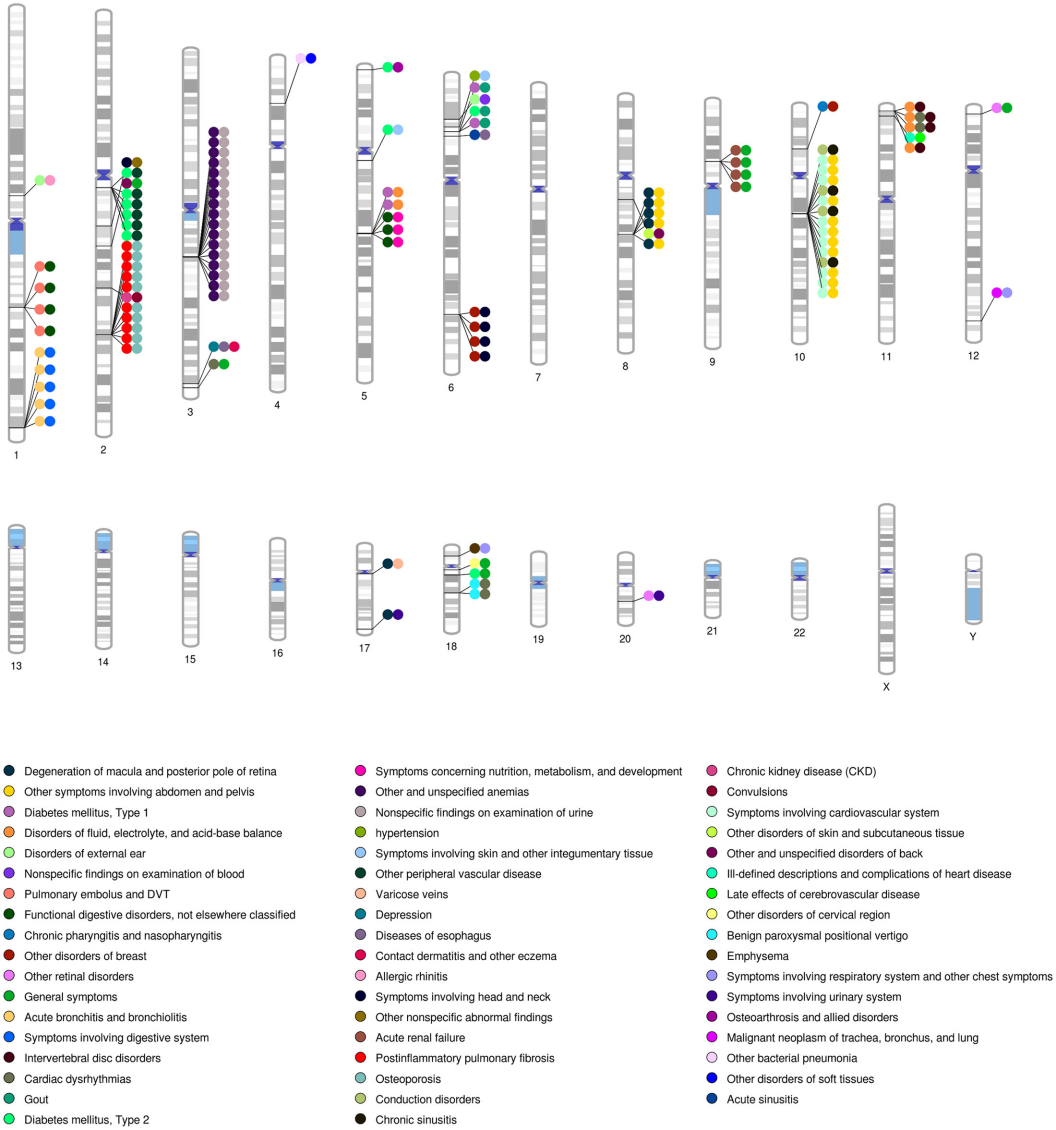
Pleiotropy: SNPs Associated with more than One Phenotype and Replicating across more than One Study for the Same ICD-9 Category. This chromosomal ideogram has lines indicating the location of the SNP, with filled colored circles indicating different ICD-9 code diagnoses associated with that particular SNP. When there are multiple pairs of the same phenotypes in the same region, this indicates regions where several SNPs in close proximity were associated with the same pairs of phenotypes.

With the wide range of phenotypes explored in PheWAS, SNPs associated with more than one phenotype can be identified, indicating potential pleiotropy. In this study, 107 out of 2,770 SNPs had associations meeting our PheWAS criteria for the same ICD-9 code category that demonstrated potential pleiotropy. An instance of such potential pleiotropy is seen with SNP rs114369580 which was found to be associated with immune related disorders T1DM and gout. The plot on the results of the SNPs associated with more than one phenotype is shown in Fig 4.

**Fig 5:**
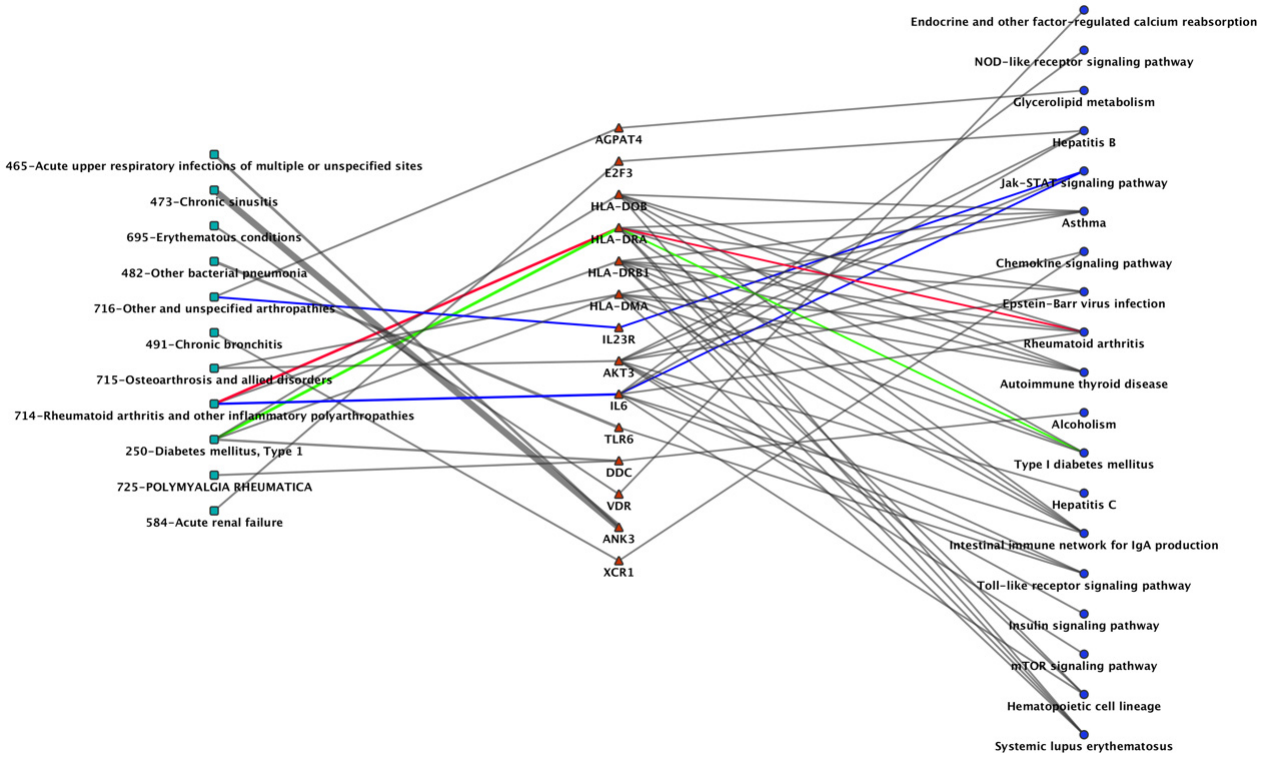
Cytoscape Network Showing the Connections between Phenotypes, the Genes with SNPs, and Pathways. In this network, green squares represent phenotype; red triangles represent genes; and blue circles are KEGG pathways. The colored lines highlight the link between phenotype and pathway. For the gene *HLA-DRA* with SNPs associated with “*714*: *rheumatoid arthritis*” and “*250*: *type 1 diabetes*” is present in the KEGG pathway of “*rheumatoid arthritis*” (red line) and “*type 1 diabetes”* (green line) respectively. Also, the blue edge shows the connection between *“714*: *rheumatoid arthritis”*, *“716*: *other specified arthropathies”* and the KEGG “*JAK-STAT signaling pathway*” through two interleukin genes, *IL23R* and *IL6*.

We further explored the interrelations between association results as a network using Cytoscape. Fig 5 shows a sub-network of our results for potential pleiotropy. We used SNPs that met our PheWAS criteria for replication for the same ICD-9 code category, and annotated those SNPs with the nearest gene. Next we used Cytoscape to link together ICD-9 codes with genes, where those ICD-9 codes are associated with the SNPs within those genes. Next we annotated the genes using the KEGG [36] pathways, and added them to the network. In the network, the diagnoses of RA and T1DM link to *HLA-DRA*, a gene found in the rheumatoid arthritis and type I diabetes KEGG disease pathways. Another interesting pattern was between *IL6* (rs2069843, rs2069849, rs1548216, rs2069844) and RA (ICD-9: 714) and *IL23R* (rs10889675) and “arthropathy” (ICD-9: 716), where both genes are from the interleukin gene family and found in a JAK-STAT signaling pathway. In Fig 5, the number of replicating association SNPs within a gene is represented by thickness of the lines. We identified that *ANK3* has most number of SNPs (a total of 65 SNPs) associated with chronic sinusitis (ICD-9: 473), Acute upper respiratory infections (ICD-9: 465).

## Discussion

Using PheWAS we found a series of associations between SNPs within or in close proximity of genes with known involvement in the immune system, such as genes within the HLA locus, including genetic variants within *HLA-DRA* associated with a series of immune-relevant diagnoses. As a member of the HLA class of molecules, *HLA-DRA* is expressed in various antigen presenting cells, and has been implicated in both T1DM [50] and RA [51].

Four SNPs in both biorepositories with associations with RA are within *IL6*. The product of the *IL6* gene is an interleukin, both a pro-inflammatory cytokine and an anti-inflammatory myokine, with an important role in regulation of inflammation and hematopoiesis. Therapies targeting the IL6 signaling system have been found effective for the treatment of RA [52].

A different group of three SNPs associated with RA mapped to *TNIP1*, a gene encoding an A20-binding protein that has a role in autoimmunity through the regulation of NFκB activation. Previous studies have shown genetic variants in *TNIP1* associated with various autoimmune conditions including psoriasis, and systemic lupus erythematous [53].

The most significant association in MyCode^®^ that replicated in BioVU was for the SNP rs6682179 mapped to the *F5* gene, which was associated with pulmonary embolus and deep vein thrombosis (ICD-9 code category 453). The *F5* gene encodes the coagulation factor V protein, a protein that circulates in the blood and is part of the blood coagulation cascade. The *F5* gene has many known mutations causing different blood coagulation disorders like factor V deficiency [54] and factor V Leiden thrombophilia [55]. We found that rs6682179 from our study is in linkage disequilibrium with rs2420371 and rs1018827 that have known associations with plasma levels of natural anticoagulant inhibitors [56] and venous thrombosis [57], respectively.

Within our replicating results, a majority of the SNPs associated with chronic sinusitis (ICD-9: 473), and acute upper respiratory infection (ICD-9: 465) phenotypes map to genes that encode members of the ankyrin protein family. Specifically, several SNPs mapping to *ANK3* are highly represented in both the acute respiratory infection and the chronic sinusitis association results, and variants mapping to *ANKS1A* were associated with acute sinusitis. Ankyrin proteins are involved in cell migration and in mediating the attachment of proteins to the cytoskeleton. While this protein family is not implicated in immune-related disorders, it is of interest that related phenotypes are associated with SNPs mapping to the same class of genes.

While we were able to seek replication of association results across two separate EHRs, a challenge was the limited overlap between the two datasets for specific ICD-9 codes as well as ICD-9 categories. Thus, it will be worthwhile to seek replication in other data sets in the future, such as through other sites of eMERGE [58,59], or evaluate the results using another method such as permutation testing. This lack of overlap is partially due to the variation in coding practices for ICD-9 codes from medical institution to institution. The lack of overlap is also likely partly due to the targeted recruitment of individuals with specific diseases at Geisinger, as the MyCode^®^ dataset is enriched for patients with obesity or AAA.

Another potential limitation in PheWAS is the multiple hypothesis-testing burden. We contrasted the significance of our results with a Bonferroni correction. We have correlated SNPs, and we also have correlated phenotypes within this study. The use of ICD-9 codes for case/control status is less well powered than traditional GWAS, due to lower case numbers. Also, the goal of PheWAS is to be exploratory, generating new hypothesis for further research. Thus we focused our evaluation within this manuscript on replication (when possible) across the two datasets and performed a meta-analysis for the results replicating across the two studies. We evaluated the SNP-phenotype associations by calculating individual SNP-phenotype associations; a future direction is evaluation and use of methods that combine information from multiple phenotypes for statistical testing.

We focused our associations on genetic variants within genes with evidence of involvement in autoimmunity and the immune system. We could have used a more general approach, seeking associations between autoimmune- and immune-traits and genome-wide SNPs, but this would have increased our multiple hypothesis testing further. Our more narrow search space will have missed genetic variants without previous evidence of association with autoimmunity and the immune system.

Our PheWAS analysis pipeline replicated previously published associations between SNPs and immune phenotypes. We also have identified a series of potential novel associations, and some of these results replicated with exact ICD-9 code or ICD-9 code category across two separate EHRs. Further studies are needed to confirm the biological validity of our potentially novel associations. Our results demonstrate potential pleiotropy, through cross-phenotype associations where individual SNPs are associated with more than one diagnosis. Further, our results show associations between inflammation/autoimmune related SNPs and disease/outcomes such as obesity, underscoring the impact of variation in the immune system on complex traits beyond direct connections to autoimmunity and the immune system.

## Supporting Information

S1 Fig Study design and analysis workflow. (TIFF)

S1 Table A list of immune related genes. (XLS)

S2 Table Results unique to each study with p < 1x10^-4^. (XLS)

S3 Table Replicating Exact ICD-9 code results. (XLSX)

S4 Table Replicating Category ICD-9 code results. (XLSX)

S5 Table Autoimmune/Immune-related category ICD-9 code results. (XLS)

S6 Table Annotation via SCAN and HaploReg. (XLS)
